# Pivotal role of High-Mobility Group Box 2 in ovarian folliculogenesis and fertility

**DOI:** 10.1186/s13048-022-01071-4

**Published:** 2022-12-20

**Authors:** Shinichiro Shirouzu, Naohiro Sugita, Narantsog Choijookhuu, Yu Yamaguma, Kanako Takeguchi, Takumi Ishizuka, Mio Tanaka, Fidya Fidya, Kengo Kai, Etsuo Chosa, Yoshihiro Yamashita, Chihiro Koshimoto, Yoshitaka Hishikawa

**Affiliations:** 1 Department of Anatomy, Histochemistry and Cell Biology, Faculty of Medicine, University of Miyazaki, 5200, 889-1692 Kihara, Kiyotake, Miyazaki Japan; 2 Department of Oral and Maxillofacial Surgery, Faculty of Medicine, University of Miyazaki, 5200, 889-1692 Kihara, Kiyotake, Miyazaki Japan; 3 Department of Ophthalmology, Faculty of Medicine, University of Miyazaki, 5200, 889-1692 Kihara, Kiyotake, Miyazaki Japan; 4 Division of Bio-resources, Department of Biotechnology, Frontier Science Research Center, University of Miyazaki, Kihara, Kiyotake, Miyazaki 5200, 889-1692 Japan; 5 Department of Surgery, Faculty of Medicine, University of Miyazaki, Kihara, Kiyotake, Miyazaki, 889–1692 Japan; 6 Department of Orthopaedic Surgery, Faculty of Medicine, University of Miyazaki, 5200, 889-1692 Kihara, Kiyotake, Miyazaki Japan

**Keywords:** HMGB2, ovary, folliculogenesis, fertility, HMGB1

## Abstract

**Background:**

High-Mobility Group Box 1 (HMGB1) and HMGB2 are chromatin-associated proteins that belong to the HMG protein family, and are involved in the regulation of DNA transcription during cell differentiation, proliferation and regeneration in various tissues. However, the role of HMGB2 in ovarian folliculogenesis is largely unknown.

**Methods:**

We investigated the functional role of HMGB1 and HMGB2 in ovarian folliculogenesis and fertilization using C57BL/6 wild type (WT) and HMGB2-knockout (KO) mice. Ovarian tissues were obtained from WT and HMGB2-KO mice at postnatal days 0, 3, 7, and 2, 6 months of age, then performed immunohistochemistry, qPCR and Western blotting analyses. Oocyte fertilization capability was examined by natural breeding and *in vitro* fertilization experiments.

**Results:**

In HMGB2-KO mice, ovary weight was decreased due to reduced numbers of oocytes and follicles. Natural breeding and *in vitro* fertilization results indicated that HMGB2-KO mice are subfertile, but not sterile. Immunohistochemistry showed that oocytes expressed HMGB2, but not HMGB1, in neonatal and adult WT ovaries. Interestingly, in HMGB2-KO ovaries, a compensatory increase in HMGB1 was found in oocyte nuclei of neonatal and 2-month-old mice; however, this was lost at 6 months of age.

**Conclusions:**

The depletion of HMGB2 led to alterations in ovarian morphology and function, suggesting that HMGB2 plays an essential role in ovarian development, folliculogenesis and fertilization.

**Supplementary Information:**

The online version contains supplementary material available at 10.1186/s13048-022-01071-4.

## Background

The ovarian follicle is the structural and functional unit of the ovary, and is composed of oocytes and supportive somatic cells, such as granulosa, theca and stromal cells [1]. Folliculogenesis begins with a dormant, primordial follicle and develops into a fully mature Graafian follicle that ovulates its oocyte into the oviduct [2]. The formation of primordial follicles starts during fetal development in humans, whereas this process occurs during neonatal development in rodents [3]. Folliculogenesis is a well-orchestrated process that is regulated by a number of genes, transcription factors and hormones [4, 5]. Recently, we found that high-mobility group box 2 (HMGB2) is an important regulator of adult mouse ovarian folliculogenesis [6]. However, the role of HMGB2 in ovarian folliculogenesis during neonatal period for murine ovarian development is largely unknown.

HMGB proteins are chromatin-associated proteins that comprise four members: HMGB1, HMGB2, HMGB3 and HMGB4 [7]. Despite HMGB group contains 4 members, highly similar structure and function are reported in HMGB1 and HMGB2, but not HMGB3 and HMGB4. The distribution pattern of HMGB1 and HMGB2 is different throughout the body [8]. The expression of HMGB1 is abundant in a variety of cells and plays an important role in the regulation of DNA transcription. In contrast, HMGB2 expression is limited to a few tissues including lymphoid tissues, liver, testis and ovary [6, 9, 10]. The differential expression of HMGB1 and HMGB2 indicates non-overlapping biological functions in various tissues. However, the functional relationship between HMGB1 and HMGB2 remains controversial. The expression of HMGB3 and HMGB4 is limited to embryo and testis, respectively. In our recent study, we demonstrated that HMGB2 is expressed in male germ cells, whereas HMGB1 is expressed in Sertoli cells. However, a compensatory increase in HMGB1 was detected in germ cells of HMGB2-KO mouse testis [11]. Therefore, the functional relationship between HMGB1 and HMGB2 requires further clarification in the mouse ovary.

Higher expression of HMGB2 is correlated with carcinogenesis, whereas it is decreased in senescent cells and adipose tissues [12–17]. HMGB2 is involved in multiple cellular events, such as gene transcription, recombination, replication, and repair processes [18]. HMGB2 binds to DNA without sequence specificity and is involved in fine-tuning gene transcription through enhancing the accessibility of transcription factors and direct protein-protein interactions [19].

In the present study, we investigated the functional role of HMGB2 in fertilization ability and ovarian folliculogenesis using WT and HMGB2-KO mice. Histomorphological changes were evaluated by hematoxylin and eosin staining. The co-localization of HMGB2 and HMGB1 in germ cells was demonstrated by immunohistochemistry in mouse ovaries during neonatal development, and 2 and 6 months of age. Finally, the reproductive ability and fertilization were examined by *in vitro* fertilization (IVF) and natural breeding experiments.

## Results

### Subfertility in HMGB2-KO mice

**Fig. 1:**
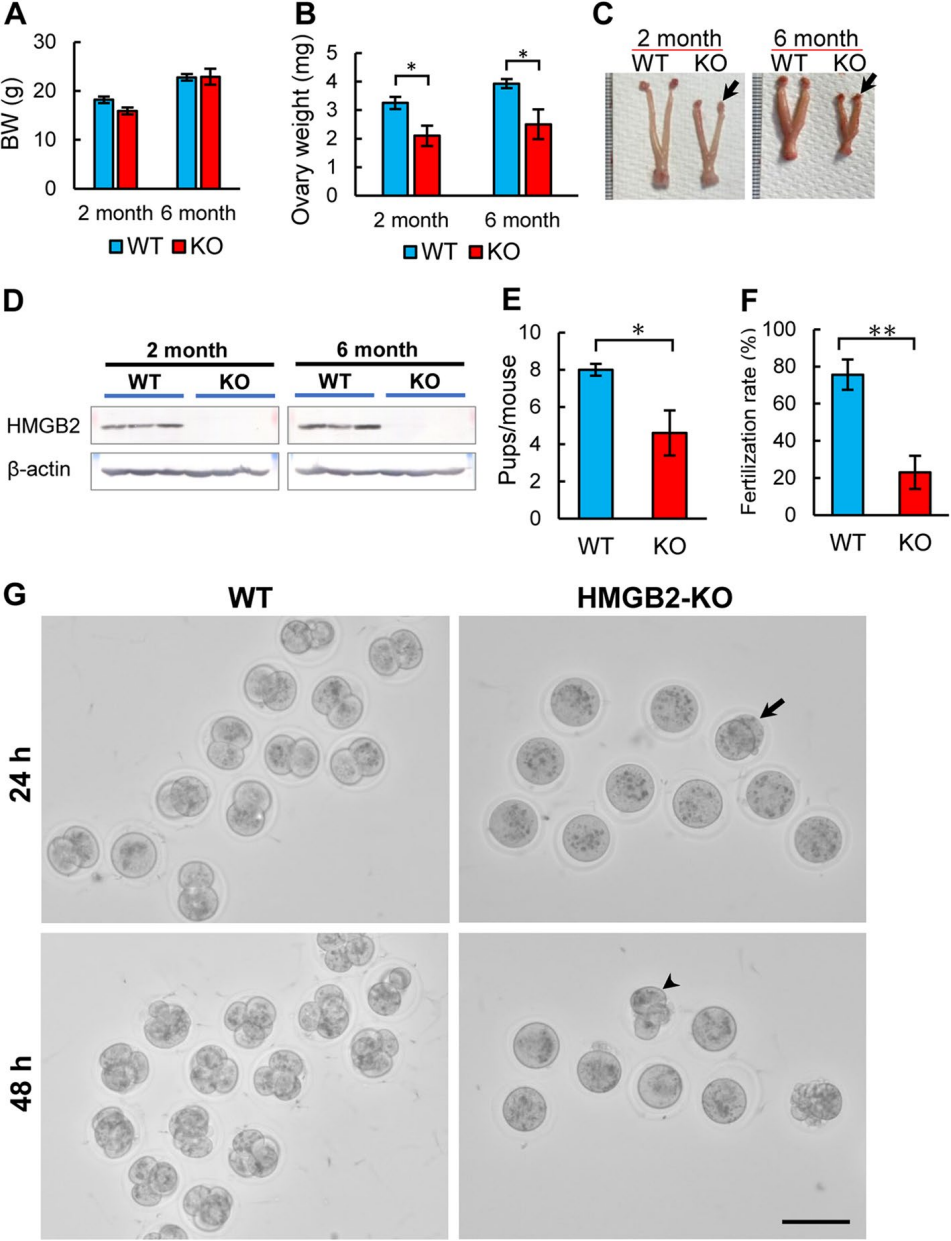
Reduced fertility in HMGB2-KO mice Body weight (**A**) and ovary weight (**B**) of 2- and 6-month-old WT and HMGB2-KO mice. Macrophotography of WT and HMGB2-KO mouse ovaries at 2 and 6 months of age (**C**). Arrows indicate ovaries of HMGB2-KO mouse. Western blotting of HMGB2 in mouse ovaries at 2- and 6-months-old WT and KO (**D**). Number of pups from WT and HMGB2-KO mice (**E**). The average number of fertilized oocytes in WT and HMGB2-KO mice (**F**). IVF using oocytes obtained from WT and HMGB2-KO mice at 24 and 48 h (**G**). Successfully fertilized oocytes at 24 and 48 h obtained from HMGB2-KO mice are indicated by an arrow and arrowhead, respectively. *P < 0.05, **P < 0.01 Student’s t-test (two tailed). Scale bar 100 µm.

HMGB2-KO mice grew normally with body weight and other phenotypes being indistinguishable from their WT-littermates at the ages of 2 and 6 months (Fig. 1A). Interestingly, ovarian weight (Fig. 1B) and size (Fig. 1C) were significantly smaller in HMGB2-KO mice at 2 and 6 months. The efficiency of HMGB2-KO was examined by western blotting analysis (Fig. 1D). To better understand ovarian function, we compared natural breeding in WT and HMGB2-KO mice. Eight-week-old WT and HMGB2-KO female mice were mated with WT male mice. A total of 40 pups were delivered in 5-WT-litters, whereas 23 pups were delivered in 5-litters of HMGB2-KO mice; which represents a significant decrease in the number of pups (Fig. 1E). Moreover, we examined fertilization ability of oocytes using *in vitro* fertilization. Successfully fertilized oocytes achieve the 2- and 4-cell stages at 24 and 48 h, respectively. The depletion of HMGB2 significantly reduced the fertilization rate of oocytes compared to WT littermates (Fig. 1F, G). Taken together, these results indicate that HMGB2 plays pivotal role in oocyte fertilization.

### Morphological changes in HMGB2-KO mouse ovary

**Fig. 2:**
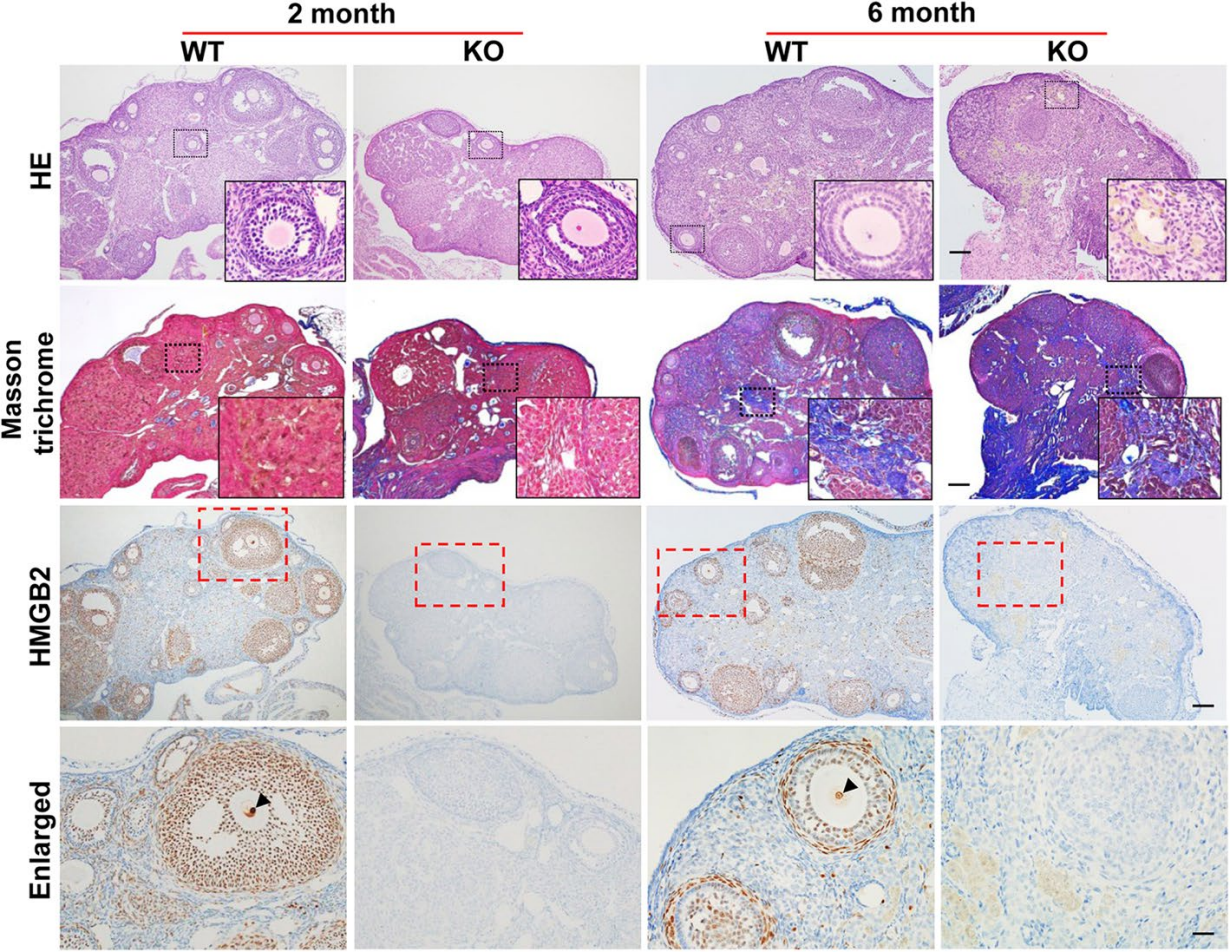
HMGB2 expression in mouse ovaries Paraffin-embedded sections of WT and HMGB2-KO mouse ovaries were used for HE and Masson trichrome staining. Boxed areas were enlarged and revealed as insets. Scale bar 100 µm. Immunohistochemistry for HMGB2 in WT and HMGB2-KO mouse ovary sections. Boxed areas were enlarged in the bottom panel. The arrowheads indicate HMGB2-positive oocytes. Scale bar 100 µm in low magnification and 20 µm in high magnification.

To understand the mechanisms underlying the observed reduced fertility, we performed histomorphological analysis of the HMGB2-KO mouse ovary. Hematoxylin and eosin-staining demonstrated that normal folliculogenesis occurred in WT mouse ovary at 2 and 6 months of age (Fig. 2). However, the number of follicles was markedly reduced in HMGB2-KO mouse ovaries than in age-matched WT ovaries. Masson trichrome staining demonstrated that fibrotic changes were not observed in either WT or HMGB2-KO mouse ovaries at 2 months. However, more fibrosis was found in 6-month-old HMGB2-KO mouse ovaries compared to age-matched WT-littermates. The expression of HMGB2 was then examined by immunohistochemistry. In WT mouse ovaries, HMGB2 was expressed in granulosa cells and theca cells of the follicles, as well as in some cells in the corpus lutea and stroma. Moreover, strong expression was observed in oocyte nuclei (Fig. 2; arrowheads). No HMGB2 staining was observed in HMGB2-KO mouse ovaries, which confirmed the efficient depletion of the target gene.

### Reduced folliculogenesis in HMGB2-KO mouse ovary

**Fig. 3:**
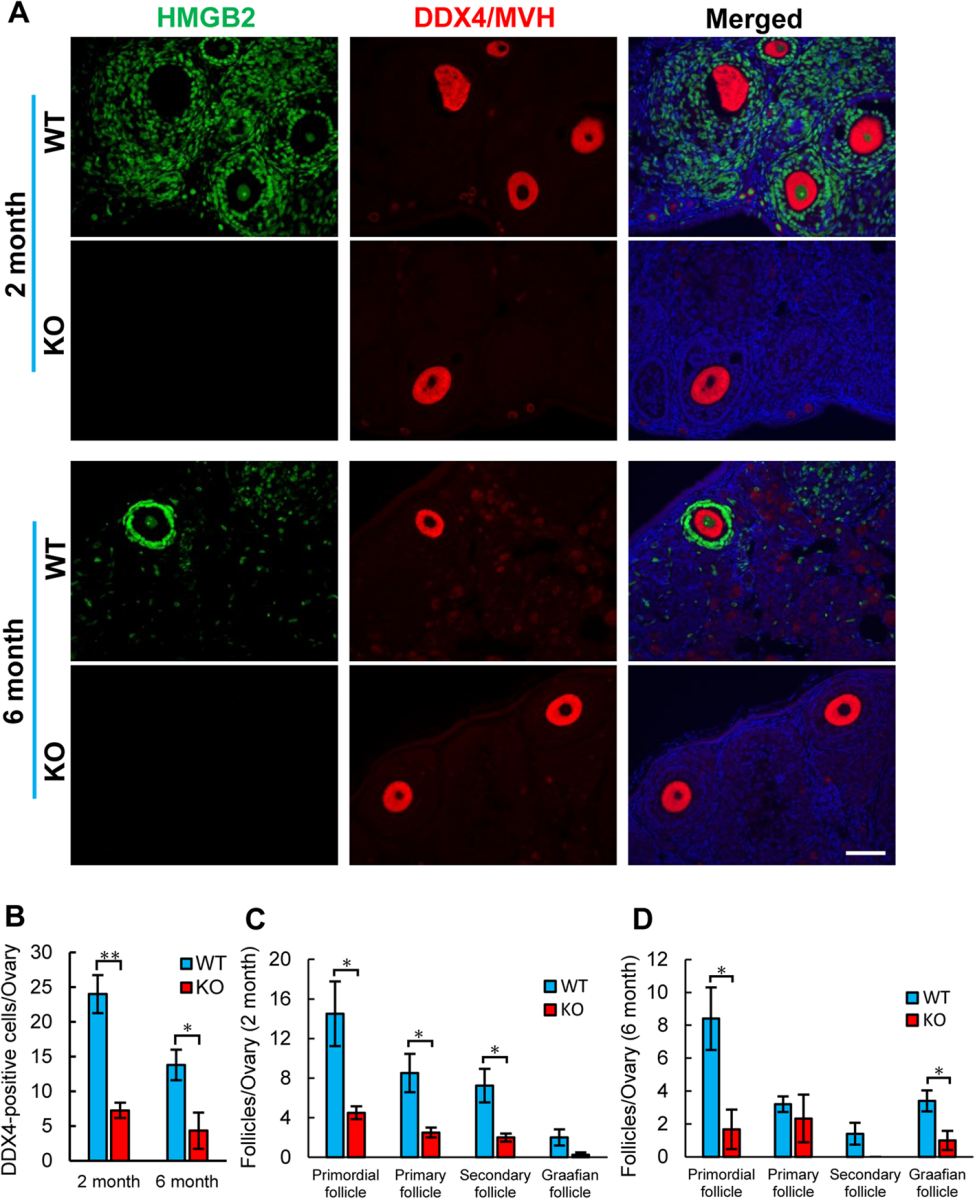
The number of germ cells and follicles Double immunofluorescence for HMGB2 (green) and DDX4/MVH (red) in paraffin-embedded ovary sections of WT and HMGB2-KO mice (**A**). The number of DDX4/MVH-positive cells in the WT and HMGB2-KO mouse ovaries (**B**). The total number of primordial, primary, secondary and Graafian follicles in 2- (**C**) and 6-month-old (**D**) WT and HMGB2-KO mouse ovaries. *P < 0.05, **P < 0.01, Student’s t-test (two tailed). Scale bar 50 µm.

The decreased ovarian size and weight in HMGB2-KO mice may be the result of reduced folliculogenesis. To confirm this hypothesis, the number of oocytes was examined by immunofluorescence using the germ cell marker DDX4/MVH (Fig. 3A). The results indicated that the number of DDX4/MVH-positive cells was significantly reduced in HMGB2-KO mouse ovaries compared to age matched WT-littermates (Fig. 3B). In the HMGB2-KO mouse ovary, the number of follicles was decreased at each stage of development, such as primordial, primary, secondary and Graafian follicles at 2 and 6 months of age (Fig. 3C, D).

### Primordial germ cells during neonatal development

**Fig. 4:**
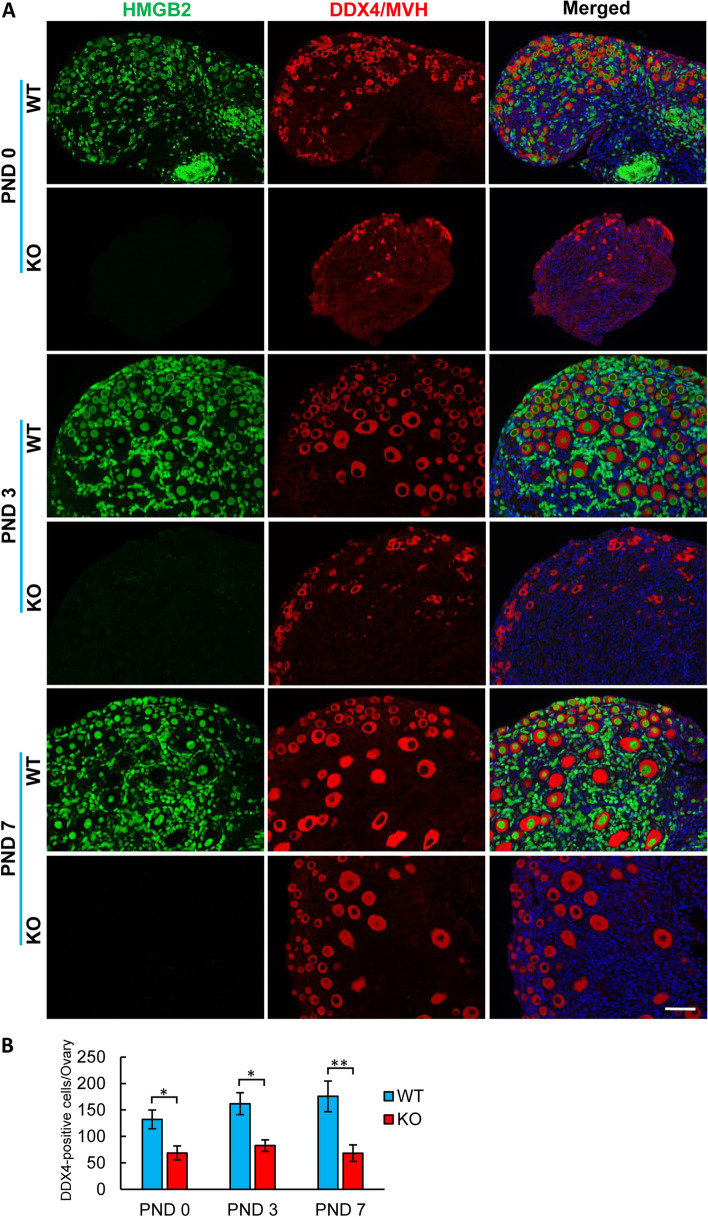
HMGB2 expression in neonatal mouse germ cells Double immunofluorescence of HMGB2 (green) and DDX4/MVH (red) in paraffin-embedded ovary sections of WT and HMGB2-KO mice at PNDs 0, 3 and 7 (**A**). The number of DDX4/MVH-positive cells in WT and HMGB2-KO mouse ovaries at PNDs 0, 3 and 7 (**B**). *P < 0.05, **P < 0.01, Student’s t-test (two tailed). Scale bar 50 µm.

Next, we studied the reason for decreased germ cell numbers in HMGB2-KO mouse ovaries. It was reported that the number of oocytes significantly decreases when cyst breakdown occurs during neonatal development [20]. Therefore, we examined the number of primordial germ cells before and after cyst breakdown, namely postnatal days (PNDs) 0, 3 and 7. In WT mouse ovary, DDX4/MVH-positive germ cells were co-localized with HMGB2 (Fig. 4A). In HMGB2-KO mouse ovary, the number of primordial germ cells was significantly decreased at PNDs 0, 3 and 7 (Fig. 4B). Thus, the depletion of HMGB2 might affect primordial germ cell apoptosis during neonatal development. To evaluate germ cell apoptosis, we examined TUNEL staining in WT and HMGB2-KO mouse ovaries at PNDs 0, 3 and 7 (Supplementary Fig. 1). However, no significant difference in germ cell apoptosis between WT and HMGB2-KO neonatal mouse ovaries was observed.

### Depletion of HMGB2 induces a compensatory increase in HMGB1 in oocytes

**Fig. 5:**
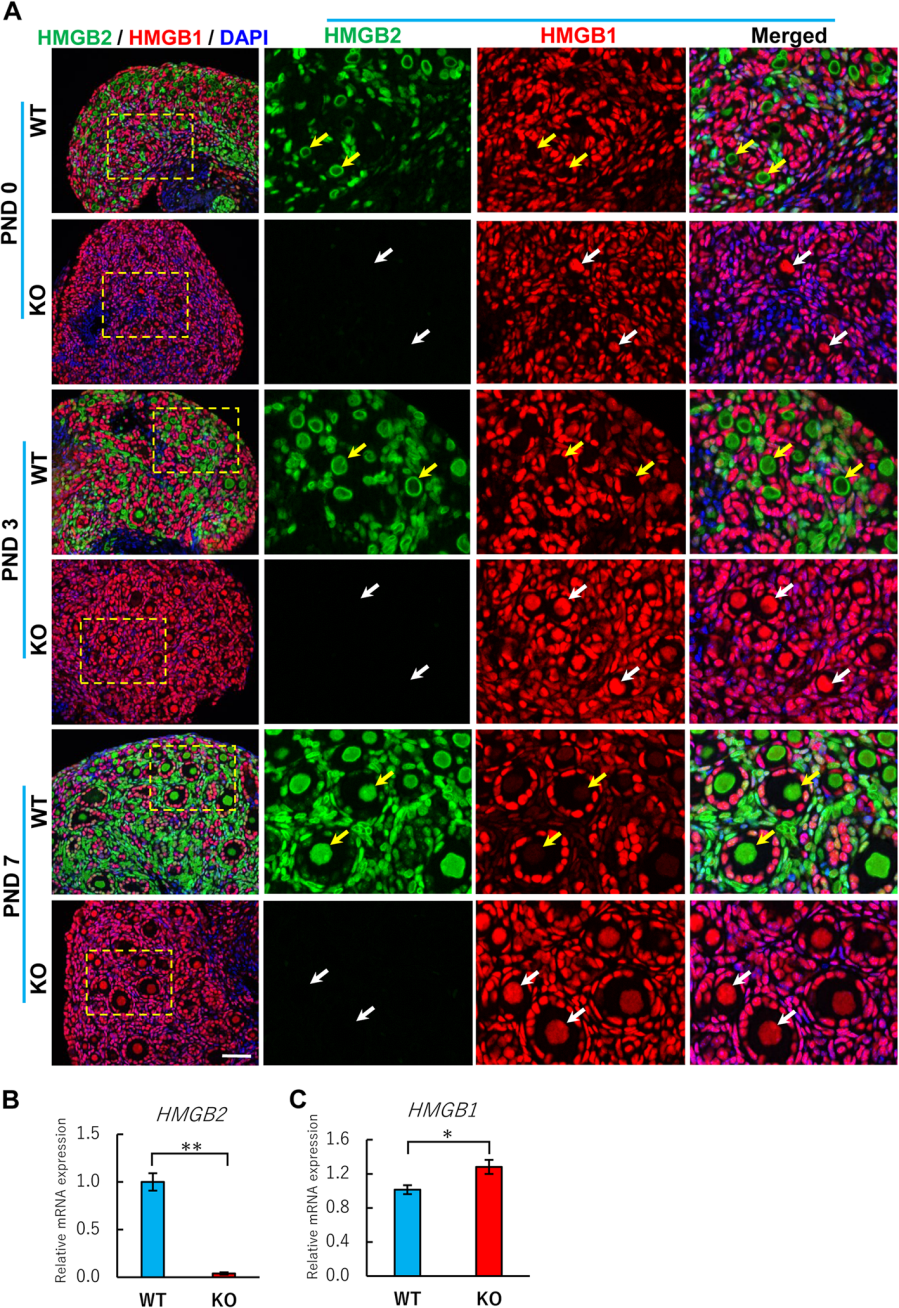
HMGB1 expression in neonatal mouse germ cells Double immunofluorescence of HMGB2 (green) and HMGB1 (red) in paraffin-embedded ovary sections of WT and HMGB2-KO mice at PNDs 0, 3 and 7 (**A**). Boxed areas were enlarged in the right panel. In WT mouse ovary, all germ cells expressed HMGB2, but not HMGB1 (yellow arrows). In HMGB2-KO mouse ovary, HMGB1 was expressed in germ cells (white arrows). Scale bar 50 µm. qPCR analysis of *HMGB2* (**B**) and *HMGB1 mRNA* (**C**) in WT and HMGB2-KO mice at PND 7.

**Fig. 6:**
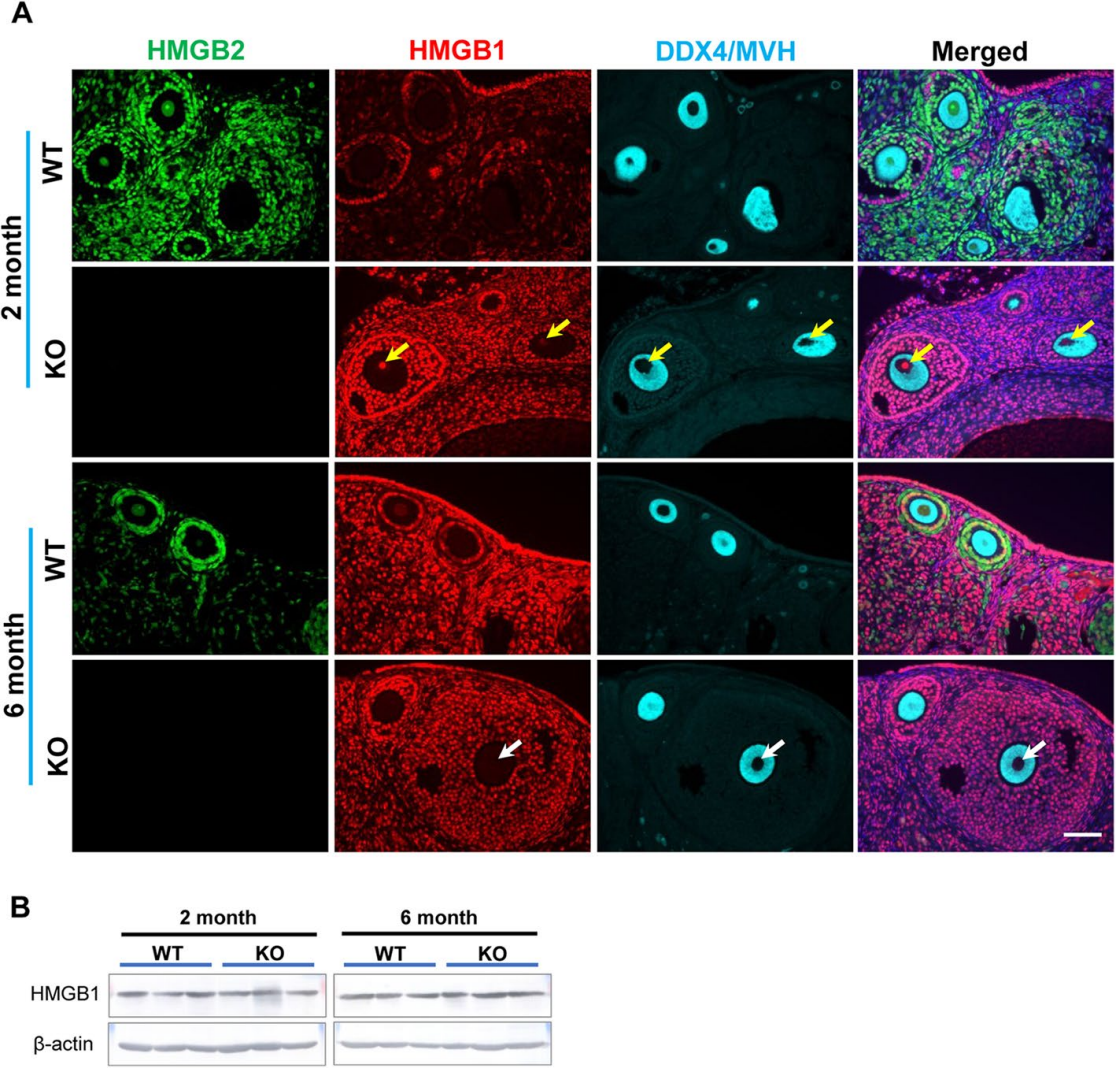
Expression of HMGB2 and HMGB1 in oocytes Immunofluorescence for HMGB2 (green), HMGB1 (red), and DDX4/MVH (blue) in paraffin-embedded ovary sections of WT and HMGB2-KO mice at 2 and 6 months of age (**A**). In 2-month-old HMGB2-KO mouse ovary, HMGB1 was expressed in oocyte nuclei (yellow arrows). In 6-month-old HMGB2-KO mouse ovary, HMGB1 expression was depleted (white arrows). Scale bar 50 µm. Western blotting of HMGB1 in mouse ovaries at 2- and 6-months-old WT and HMGB2-KO (**B**).

The co-localization of HMGB2 and HMGB1 was examined by double immunofluorescence in neonatal mouse ovaries (Fig. 5A). In WT mouse ovaries, primordial germ cells expressed HMGB2, but not HMGB1 (yellow arrows). Interestingly, HMGB1 was expressed in primordial germ cells in HMGB2-KO mouse ovaries at PNDs 0, 3 and 7 (white arrows). These results indicate that the depletion of HMGB2 induces a compensatory increase in HMGB1 in primordial germ cells during neonatal development. In addition, the relative expressions of *HMGB2* and *HMGB1* mRNAs were examined by qPCR. In PND 7 mouse ovary, the *HMGB2* mRNA expressed in WT, but almost no expression was detected in KO mice ovaries (Fig. 5B). The significant increase in *HMGB1* mRNA expression was detected in HMGB2-KO mice ovaries (Fig. 5C). Furthermore, we examined the co-localization of HMGB2 and HMGB1 in adult mouse germ cells (Fig. 6A). In WT mouse ovaries at 2 and 6 months of age, oocytes expressed HMGB2, but not HMGB1, as with neonatal mice. In HMGB2-KO mouse ovaries, the compensatory increase in HMGB1 remained at 2 months of age (yellow arrows). Unexpectedly, HMGB1 was not detected in oocytes of 6-month-old HMGB2-KO mouse ovaries (white arrows), which suggests that the compensatory increase in HMGB1 is lost as the age of the mouse progresses. Western blotting analysis revealed that the HMGB1 protein expression was similar in WT and HMGB2-KO mice ovaries at 2- and 6-months-old (Fig. 6B). Raw data revealed as supplementary figure s2.

## Discussion

In the present study, the major findings were that depletion of HMGB2 led to functional and morphological changes in ovaries, such as subfertility and defective folliculogenesis. In the HMGB2-KO mouse ovary, a compensatory increase in HMGB1 was detected in oocyte nuclei until 2 months of age; however, HMGB1 expression is subsequently lost in 6-month-old mice. These results indicate that HMGB2 plays pivotal role in mouse folliculogenesis and fertilization.

Despite the gross appearance and growth of HMGB2-KO mice being indistinguishable from WT-littermates, ovarian morphology and function were affected, namely decreased ovarian size and weight, and germ cell number. The results of the natural breeding study revealed that significantly fewer pups were born to HMGB2-KO female mice than age-matched WT-littermates. Moreover, IVF of HMGB2-KO oocytes resulted in significantly fewer fertilized oocytes than with WT oocytes. Altogether, these results indicate that HMGB2-KO mice are subfertile, but not sterile. To unravel the underlying molecular mechanism, we performed detailed histomorphological analysis. The intense staining of HMGB2 in the nuclei of oocytes, granulosa and theca cells in WT mouse ovaries indicates that it plays an important role in oocyte and follicle development.

The depletion of HMGB2 led to a decreased number of oocytes and folliculogenesis, suggesting an important role in ovarian function. A similar subfertile phenotype with altered folliculogenesis was observed in androgen receptor-depleted (AR^−/−^) mice [21]. While the general phenotype of AR^−/−^ mice appears normal, reproductive function was altered, including longer estrous cycle, reduction in the number of corpora lutea and decreased expression of progesterone receptor. Moreover, retarded oocyte growth and follicular development was observed in Foxo3 transgenic mice [22]. Increased apoptotic germ cells were reported in HMGB2-KO male mice [10, 11]. In normal ovarian development, germ cell number is decreased dramatically through apoptosis during cyst breakdown [23, 24]. Therefore, we hypothesized that germ cell apoptosis may be elevated during cyst breakdown in HMGB2-KO mouse. In fact, TUNEL analysis revealed that the number of apoptotic cells was similar in WT and HMGB2-KO mouse ovaries during neonatal development. The number of germ cells were significantly decreased at PNDs 0, 3 and 7; however, the germ cells were not affected by apoptosis, suggesting that germ cells were already decreased before birth. The number of oocytes was consistently fewer in neonatal and adult HMGB2-KO mouse ovaries, indicating that the number of oocytes did not fluctuate. IVF results suggest that the reduced fertilization ability in oocytes that obtained from HMGB2-KO mice. Moreover, the number of oocytes was significantly decreased in HMGB2-KO mice. Therefore, we hypothesized that either number of oocytes and fertilization ability were mainly depend upon HMGB2 expression.

A differential expression pattern for HMGB2 and HMGB1 has been observed, which indicates non-overlapping biological functions in various tissues even though there is high similarity in both sequence and structure [25]. All oocytes in WT mouse ovaries expressed HMGB2, but not HMGB1. Unexpectedly, HMGB1 was expressed in oocytes of HMGB2-KO mouse ovary, demonstrating a compensatory increase in oocytes of neonatal and adult mice. Although immunohistochemistry detected the compensatory increase in HMGB1 in oocytes of 2-month old HMGB2-KO mouse ovary, the total HMGB1 protein expression was similar with WT-littermates. These findings could be explained by HMGB1 expression in all cells of the ovary in both genotypes. The difference is undetectable due to lower ratio of germ cells/somatic cells in the adult mouse ovary. On the other hand, the ratio of oocytes/somatic cells is much higher during neonatal development, such as PND 7. Interestingly, qPCR detected the significant increase in *HMGB1* mRNA in HMGB2-KO mouse ovaries at PND 7 comparing to WT-littermates.

Recently we reported a similar finding in male reproductive tissue; a compensatory increase in HMGB1 was found in spermatocytes of HMGB2-KO mice [11]. In this study, the compensatory increase of HMGB1 in oocytes is lost at 6 months of age, suggesting that functional compensation was lost as age increased. In HMGB2-KO mouse ovaries, age-related changes begin prior to 6 months of age, such as ovarian fibrosis and shrinkage. Moreover, aging-related loss of HMGB2 induces osteoarthritis and reduces cellularity in humans [26]. In addition, the correlation of HMGB2 and aging was demonstrated by Ly et al. [27]. Interestingly, 6000 human genes were examined in aged and progeria people, and the results revealed that HMGB2 was one of 9 genes that were down-regulated. Therefore, aging related decline of reproductive function may be correlated with HMGB2 expression in ovaries.

In summary, HMGB depletion induced subfertility and decreased folliculogenesis, demonstrating the important role of HMGB2 in mouse fertility and follicle development. The number of oocytes was decreased at birth, consequently, a decreased number of germ cells was observed in adulthood. A compensatory increase in HMGB1 expression was found in HMGB2-KO mouse oocytes, which was lost at 6 months of age.

## Materials and methods

### Chemicals and biochemicals

Trizma base, bovine serum albumin (BSA), 3-aminopropyl-triethoxysilane, Tyramine hydrochloride and Brij L23 were purchased from Sigma Chemical Co. (St Louis, MO, USA). Paraformaldehyde (PFA) was purchased from Merck (Darmstadt, Germany). FITC-succinimidyl ester, rhodamine-succinimidyl ester and 4`,6-diamidino-2-phenylindole (DAPI) were purchased from Thermo Fisher (USA). 3,3`-Diaminobenzidine–4 HCl (DAB) was purchased from Dojindo Chemicals (Kumamoto, Japan). PMSG (pregnant mare serum gonadotropin) and hCG (human chorionic gonadotropin) were purchased from Aska animal health (Tokyo, Japan). All other reagents used in this study were purchased from Fujifilm Wako Pure Chemicals (Osaka, Japan). The Masson trichrome staining kit was obtained from Muto Pure Chemicals (Tokyo, Japan). All other reagents used in this study were purchased from Fujifilm Wako Pure Chemicals (Osaka, Japan).

### Animals and tissue preparation

Female C57BL/6J wildtype (WT) and HMGB2-KO mice (2 and 6 months) were used in the present study. Age-matched mice were used in this experiment, but estrus stage was not synchronized. Moreover, PND 0, 3 and 7 mice ovaries were used. The day of delivery was counted as PND 0. The derivation of genomic HMGB2-KO mouse has been described [9]. Mice were kept under constant 12 h dark/lighting condition and fed normal chow with drinking water *ad libitum*. The experimental protocol was approved by the Animal Ethics Review Committee of the University of Miyazaki. The ARRIVE Essential 10 guideline was used to formulate study design, sample preparation, result observation and data analysis. Mouse ovary was fixed in 4% PFA in phosphate-buffered saline (PBS) (pH 7.4) for overnight at room temperature (RT) and then embedded in paraffin. In each experimental group, 4–6 mice were used.

### Histological analysis

Ovarian morphology was analyzed by hematoxylin and eosin (HE) staining. Ovarian fibrosis was examined by Masson trichrome staining, according to the manufacturer’s instructions.

### Natural breeding and in vitro fertilization

Ovarian function was examined using a natural breeding study involving the mating of 8-week-old male WT mice with female WT (*n* = 5) and HMGB2-KO mice (*n* = 5). The *in vitro* fertilization study used 8-week-old WT and HMGB2-KO female mice. Superovulation was induced by intraperitoneal injection of PMSG. After 48 h, hCG was administered and the oocytes were subsequently obtained from both the right and left oviducts [28]. Spermatozoa were collected from the cauda epididymis of WT male (10–12 weeks) mice and subsequently capacitated for 1 h in 5% CO_2_ at 37°C. Spermatozoa were added to modified human tubal fluid (mHTF) medium containing oocytes. After 3 h of incubation (37°C, 5% CO_2_), spermatozoa were washed with mHTF and incubated for 48 h. The number of fertilized oocytes were counted at 24 and 48 h.

### Immunohistochemistry

Paraffin-embedded tissues were cut into 4-µm-thick sections and placed onto silane-coated slide glasses. The sections were deparaffinized with toluene and rehydrated using a graded ethanol series, then autoclaved at 120ºC for 15 min in 10 mM citrate buffer (pH 6.0) [29, 30]. After inhibition of endogenous peroxidase activity with 3% H_2_O_2_ in methanol for 30 min, the sections were pre-incubated with 500 µg/ml normal goat IgG and 1% BSA in PBS for 1 h to block non-specific binding of antibodies. The sections were then reacted with the following primary antibodies for 16–17 h: anti-HMGB2 (Abcam, ab124670), anti-HMGB1 (Abcam, ab18256), anti-DDX4/MVH (Abcam, ab13840). After washing in 0.075% Brij/PBS, the HRP-sites were visualized with DAB and H_2_O_2_ (brown) [31]. For immunofluorescence, sections were reacted with FITC-or Rhodamine-conjugated tyramide, Alexa-633 conjugated goat anti-rabbit antibody and then counterstained with DAPI [9]. As a negative control, normal rabbit IgG was used at the same concentration instead of the primary antibody in each experiment. Microphotographs were taken using Olympus DP74 camera and Olympus BX53 light microscope (cellSens Imaging Software, version 3.1). Fluorescent micrographs were taken using DAPI (excitation 360 nm), FITC (excitation 470 nm) and TRITC (excitation 545 nm) filters and Keyence BZ-X700 microscope.

### Western blot analysis

Tissues were homogenized in hot SDS lysis buffer and centrifuged at 15,000 rpm for 30 min at 4ºC [11]. Then supernatant was collected and stored at − 80ºC. The protein concentration in each preparation was determined using a BCA assay kit. Lysate containing 15 µg of protein was separated by 10% SDS-PAGE, and the proteins were electrophoretically transferred onto PVDF membranes. The membranes were blocked with 5% nonfat milk in Tris-buffered saline (TBS; 20 mM Tris buffer [pH 7.6], 150 mM NaCl) for 1 h at RT and then incubated overnight with anti-HMGB2 (1,000x), or anti-HMGB1 (1,000x) and β-actin (15,000x) antibodies with TBS/0.05% Triton X-100 buffer. As a secondary antibody, HRP-goat anti-rabbit IgG or HRP-goat anti-mouse IgG was diluted with TBS buffer for 1 h, and the membranes were washed 3 times for 10 min each with TBS/0.05% Triton X-100 buffer. Bands were visualized with DAB, Ni, Co, and H_2_O_2_. β-actin was used as an internal standard in each lane for normalization of target protein expression.

### qPCR analysis

Total RNA was extracted from snap frozen ovarian tissues using Reliprep RNA tissue Miniprep System (Promega, Madison, USA). RNA was reverse transcribed to cDNA using Moloney murine leukemia virus reverse transcriptase (Invitrogen, Carlsbad, CA, USA). Transcript expression levels were analyzed by an ABI StepOne plus Real-Time PCR System (Applied Biosystems, Waltham, MA, USA) using Fast SYBR Green (Applied Biosystems). β-actin was used for normalization and relative gene expression was calculated using the 2 − ΔΔct method. The primer pairs were listed below. HMGB2 F-5'-TCCTGGTAGGCCAACAGGCT-3' and R-5'-AGCTAATGTTGAGCTGCACTTG-3', HMGB1 F-5'- GGCTGACAAGGCTCGTTATG-3', R-5'- GGGCGGTACTCAGAACAGAA-3' and β-actin F-5'- TCCTCCCTGGAGAAGAGCTAC − 3', R-5'- TCCTGCTTGCTGATCCACAT − 3'. In each experimental group, 3–4 mice were used.

### Tunel staining

TUNEL staining was performed as described previously [11, 32]. Briefly, the sections were deparaffinized with toluene and rehydrated using a graded ethanol series. After washing with PBS, the sections were treated with 10 µg/ml of proteinase K in PBS at 37°C for 15 min. The sections were then rinsed once with distilled water and incubated with TdT buffer (25 mM Tris-HCl buffer [pH 6.6], containing 0.2 M potassium cacodylate and 0.25 mg/ml BSA) alone for 30 min at RT. The sections were then reacted with 800 U/ml TdT dissolved in a TdT buffer supplemented with 5 µM biotin-16-dUTP, 20 µM dATP, 1.5 mM CoCl_2_, and 0.1 mM dithiothreitol at 37°C for 90 min. The reaction was terminated by washing with 50 mM Tris/HCl buffer (pH 7.5) for 15 min. Endogenous peroxidase activity was inhibited by immersing the slides in 0.3% H_2_O_2_ in methanol for 15 min at RT. Signals were detected immunohistochemically using an HRP-conjugated goat anti-biotin antibody, and HRP-sites were visualized by DAB, Ni, Co, and H_2_O_2_. As a negative control, adjacent sections were subjected to reaction without TdT.

### Quantitative analysis

The number of germ cells, follicles and TUNEL-positive cells were counted in 4–6 mice per experimental group. In each mouse, one micrograph that containing whole ovarian cross section was used for quantitative analysis.

### Statistical analysis

All data are expressed as the mean ± standard error of the mean. Statistical significance was assessed using the Student’s *t*-test. *P* < 0.05 was considered statistically significant. All analyses were performed with the Statistical Package for Social Sciences (version 20; IBM Corp., Armonk, NY, USA).

## Supplementary Information

**Additional file 1**. **Figure 1**. Apoptotic germ cells in neonatal HMGB2-KO mouse **Figure 2**. Original images of western blotting that revealed in Fig. 1d and Fig. 6b. The expression of HMGB2 (A) and HMGB1 (B) were examined in 3 mice in each genotypes.

## Data Availability

The datasets used and analyzed in the current study are available from the corresponding author on reasonable request.

## References

[1] Richards JS, Pangas SA (2010). The ovary: basic biology and clinical implications. J Clin Invest.

[2] Dunlop CE, Anderson RA (2014). The regulation and assessment of follicular growth. Scand J Clin Lab Invest Suppl.

[3] Hsueh AJ, Kawamura K, Cheng Y, Fauser BC (2015). Intraovarian control of early folliculogenesis. Endocr Rev.

[4] Hirano M, Wada-Hiraike O, Fu H (2017). The Emerging Role of FOXL2 in Regulating the Transcriptional Activation Function of Estrogen Receptor β: An Insight Into Ovarian Folliculogenesis. Reprod Sci.

[5] Pangas SA, Rajkovic A (2006). Transcriptional regulation of early oogenesis: in search of masters. Hum Reprod Update.

[6] Yamaguma Y, Sugita N, Choijookhuu N (2022). Crucial role of high-mobility group box 2 in mouse ovarian follicular development through estrogen receptor beta. Histochem Cell Biol.

[7] Ueda T, Yoshida M (2010). HMGB proteins and transcriptional regulation. Biochim Biophys Acta.

[8] Stros M (2010). HMGB proteins: interactions with DNA and chromatin. Biochim Biophys Acta.

[9] Yano K, Choijookhuu N, Ikenoue M (2022). Spatiotemporal expression of HMGB2 regulates cell proliferation and hepatocyte size during liver regeneration. Sci Rep.

[10] Ronfani L, Ferraguti M, Croci L (2001). Reduced fertility and spermatogenesis defects in mice lacking chromosomal protein Hmgb2. Development.

[11] Sugita N, Choijookhuu N, Yano K (2021). Depletion of high-mobility group box 2 causes seminiferous tubule atrophy via aberrant expression of androgen and estrogen receptors in mouse testis. Biol Reprod.

[12] Chen K, Zhang J, Liang F (2021). HMGB2 orchestrates mitotic clonal expansion by binding to the promoter of C/EBPβ to facilitate adipogenesis. Cell Death Dis.

[13] Zirkel A, Nikolic M, Sofiadis K (2018). HMGB2 Loss upon Senescence Entry Disrupts Genomic Organization and Induces CTCF Clustering across Cell Types. Mol Cell.

[14] Fu D, Li J, Wei J (2018). HMGB2 is associated with malignancy and regulates Warburg effect by targeting LDHB and FBP1 in breast cancer. Cell Commun Signal.

[15] Lee D, Taniguchi N, Sato K (2018). HMGB2 is a novel adipogenic factor that regulates ectopic fat infiltration in skeletal muscles. Sci Rep.

[16] Cámara-Quílez M, Barreiro-Alonso A, Vizoso-Vázquez Á (2020). The HMGB1-2 Ovarian Cancer Interactome. The Role of HMGB Proteins and Their Interacting Partners MIEN1 and NOP53 in Ovary Cancer and Drug-Response. Cancers (Basel).

[17] Barreiro-Alonso A, Lamas-Maceiras M, Rodríguez-Belmonte E, Vizoso-Vázquez Á, Quindós M, Cerdán ME (2016). High Mobility Group B Proteins, Their Partners, and Other Redox Sensors in Ovarian and Prostate Cancer. Oxid Med Cell Longev..

[18] Bagherpoor AJ, Kučírek M, Fedr R, Sani SA, Štros M (2020). Nonhistone Proteins HMGB1 and HMGB2 Differentially Modulate the Response of Human Embryonic Stem Cells and the Progenitor Cells to the Anticancer Drug Etoposide. Biomolecules.

[19] Bianchi ME, Agresti A (2005). HMG proteins: dynamic players in gene regulation and differentiation. Curr Opin Genet Dev.

[20] Kim JY (2012). Control of ovarian primordial follicle activation. Clin Exp Reprod Med.

[21] Hu YC, Wang PH, Yeh S (2004). Subfertility and defective folliculogenesis in female mice lacking androgen receptor. Proc Natl Acad Sci U S A.

[22] Liu L, Rajareddy S, Reddy P (2007). Infertility caused by retardation of follicular development in mice with oocyte-specific expression of Foxo3a. Development.

[23] Islam MR, Ichii O, Nakamura T (2021). Developmental Changes of the Ovary in Neonatal Cotton Rat (*Sigmodon hispidu*s). Front Physiol.

[24] Suzuki H, Kanai-Azuma M, Kanai Y (2015). From Sex Determination to Initial Folliculogenesis in Mammalian Ovaries: Morphogenetic Waves along the Anteroposterior and Dorsoventral Axes. Sex Dev.

[25] Agresti A, Bianchi ME (2003). HMGB proteins and gene expression. Curr Opin Genet Dev.

[26] Taniguchi N, Caramés B, Ronfani L (2009). Aging-related loss of the chromatin protein HMGB2 in articular cartilage is linked to reduced cellularity and osteoarthritis. Proc Natl Acad Sci U S A.

[27] Ly DH, Lockhart DJ, Lerner RA, Schultz PG (2000). Mitotic misregulation and human aging. Science.

[28] Quinn P, Whittingham DG (1982). Effect of fatty acids on fertilization and development of mouse embryos in vitro. J Androl.

[29] Srisowanna N, Choijookhuu N, Yano K (2019). The Effect of Estrogen on Hepatic Fat Accumulation during Early Phase of Liver Regeneration after Partial Hepatectomy in Rats. Acta Histochem Cytochem.

[30] Choijookhuu N, Sato Y, Nishino T, Endo D, Hishikawa Y, Koji T (2012). Estrogen-dependent regulation of sodium/hydrogen exchanger-3 (NHE3) expression via estrogen receptor β in proximal colon of pregnant mice. Histochem Cell Biol.

[31] Batmunkh B, Choijookhuu N, Srisowanna N (2017). Estrogen Accelerates Cell Proliferation through Estrogen Receptor α during Rat Liver Regeneration after Partial Hepatectomy. Acta Histochem Cytochem.

[32] Liu J, Zhang W, Wu Z, Dai L, Koji T (2018). Changes in DNA Methylation of Oocytes and Granulosa Cells Assessed by HELMET during Folliculogenesis in Mouse Ovary. Acta Histochem Cytochem.

