# Correction: Pivotal role of High-Mobility Group Box 2 in ovarian folliculogenesis and fertility

**DOI:** 10.1186/s13048-023-01105-5

**Published:** 2023-01-24

**Authors:** Shinichiro Shirouzu, Naohiro Sugita, Narantsog Choijookhuu, Yu Yamaguma, Kanako Takeguchi, Takumi Ishizuka, Mio Tanaka, Fidya, Kengo Kai, Etsuo Chosa, Yoshihiro Yamashita, Chihiro Koshimoto, Yoshitaka Hishikawa

**Affiliations:** 1 Department of Anatomy, Histochemistry and Cell Biology, Faculty of Medicine, University of Miyazaki, 5200, 889-1692 Kihara, Kiyotake, Miyazaki Japan; 2 Department of Oral and Maxillofacial Surgery, Faculty of Medicine, University of Miyazaki, 5200, 889-1692 Kihara, Kiyotake, Miyazaki Japan; 3 Department of Ophthalmology, Faculty of Medicine, University of Miyazaki, 5200, 889-1692 Kihara, Kiyotake, Miyazaki Japan; 4 Division of Bio-resources, Department of Biotechnology, Frontier Science Research Center, University of Miyazaki, Kihara, Kiyotake, Miyazaki 5200, 889-1692 Japan; 5 Department of Surgery, Faculty of Medicine, University of Miyazaki, Kihara, Kiyotake, Miyazaki 889–1692 Japan; 6 Department of Orthopaedic Surgery, Faculty of Medicine, University of Miyazaki, 5200, 889-1692 Kihara, Kiyotake, Miyazaki Japan

**Correction: J Ovarian Res 15:133 (2022)**10.1186/s13048-022-01071-4

Following publication of the original article [1], the authors identified an error in authorgroup. Author name Fidya Fidya should be Fidya. The original article has been corrected.
