# Supplementary material for: Pivotal role of High-Mobility Group Box 2 in ovarian folliculogenesis and fertility
Source: J Ovarian Res. 2022 Dec 20;15:133. doi: 10.1186/s13048-022-01071-4 (PMC9769043; doi:10.1186/s13048-022-01071-4)
Supplement: Supplementary file 1 — Additional file 1. Figure 1. Apoptotic germ cells in neonatal HMGB2-KO mouse Figure 2. Original images of western blotting that revealed in Fig. 1d and Fig. 6b. The expression of HMGB2 (A) and HMGB1 (B) were examined in 3 mice in each genotypes. [file 13048_2022_1071_MOESM1_ESM.pdf]

Pivotal role of High-Mobility Group Box 2 in ovarian folliculogenesis and fertility

Shinichiro Shirouzu[1, 2, ‡], Naohiro Sugita[1, 3, ‡], Narantsog Choijookhuu[1], Yu Yamaguma[1, 2], Kanako Takeguchi[4], Takumi Ishizuka[1], Mio Tanaka[1], Fidya Fidya[1], Kengo Kai[1, 5], Etsuo Chosa[6], Yoshihiro Yamashita[2], Chihiro Koshimoto[4] and Yoshitaka Hishikawa[1]

[1]Department of Anatomy, Histochemistry and Cell Biology, Faculty of Medicine, University of Miyazaki, 5200 Kihara, Kiyotake, Miyazaki 889-1692, Japan

[2]Department of Oral and Maxillofacial Surgery, Faculty of Medicine, University of Miyazaki, 5200 Kihara, Kiyotake, Miyazaki 889-1692, Japan

[3]Department of Ophthalmology, Faculty of Medicine, University of Miyazaki, 5200 Kihara, Kiyotake, Miyazaki 889-1692, Japan

[4]Division of Bio-resources, Department of Biotechnology, Frontier Science Research Center, University of Miyazaki, Kihara 5200 Kihara, Kiyotake, Miyazaki 889-1692, Japan

[5]Department of Surgery, Faculty of Medicine, University of Miyazaki, Miyazaki 889-1692, Japan

[6]Department of Orthopaedic Surgery, Faculty of Medicine, University of Miyazaki, 5200 Kihara, Kiyotake, Miyazaki 889-1692, Japan

[‡]These authors contributed equally to this study.

Running title: The role of HMGB2 in ovarian folliculogenesis and fertility.

Correspondence:

Narantsog Choijookhuu, MD., Ph.D.,

Department of Anatomy, Histochemistry and Cell Biology, Faculty of Medicine, University of Miyazaki, 5200 Kihara, Kiyotake, Miyazaki 889-1692, Japan. Tel: +81-985-85-1783. Fax: +81-985-85-9851 E-mail: narantsog@med.miyazaki-u.ac.jp

Supplementary figure 1

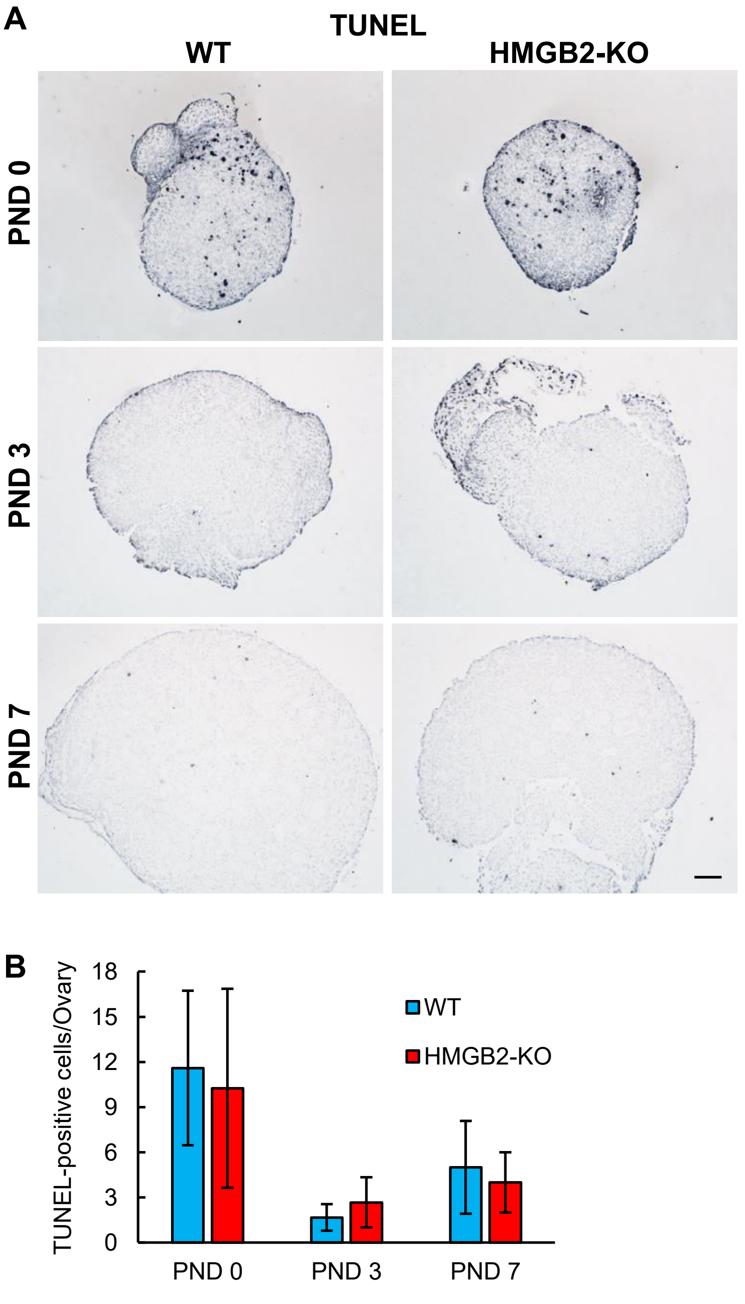

Supplementary fig. 1. Apoptotic germ cells in neonatal HMGB2-KO mouse ovary.

WT and HMGB2-KO mouse ovary sections were analyzed by TUNEL at PNDs 0, 3 and 7 (**A**). The number of TUNEL-positive cells in WT and HMGB2-KO mouse ovaries at PNDs 0, 3 and 7 (**B**). Scale bar 50 μm.

Supplementary figure 2

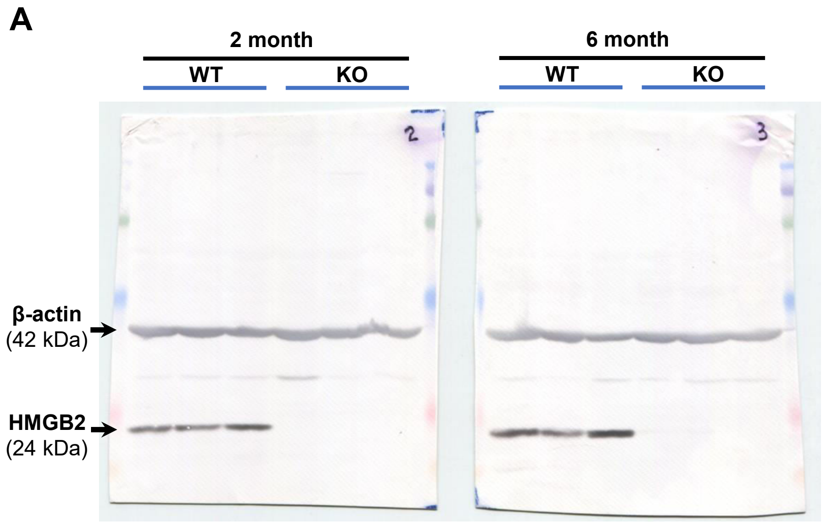

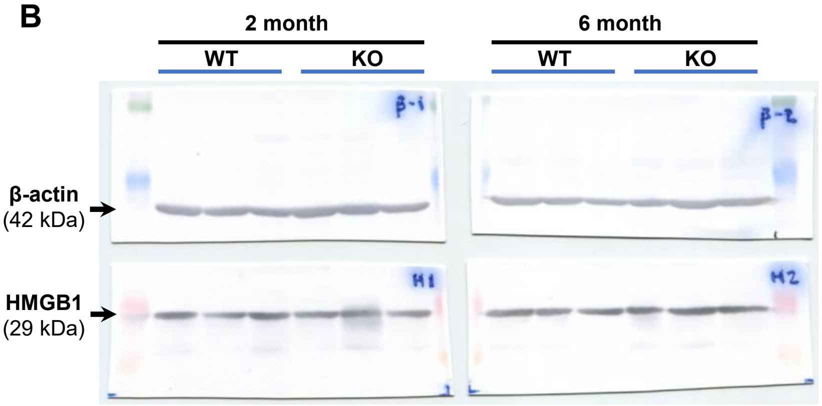

Supplementary fig. 2.
Original images of western blotting that revealed in Fig. 1d and Fig. 6b. The expression of HMGB2 (**A**) and HMGB1 (**B**) were examined in 3 mice in each genotypes.
